# Longitudinal Studies on Alzheimer Disease Mouse Models with Multiple Tracer PET/CT: Application of Reduction and Refinement Principles in Daily Practice to Safeguard Animal Welfare during Progressive Aging

**DOI:** 10.3390/ani13111812

**Published:** 2023-05-30

**Authors:** Giovanna Palumbo, Lea Helena Kunze, Rosel Oos, Karin Wind-Mark, Simon Lindner, Barbara von Ungern-Sternberg, Peter Bartenstein, Sibylle Ziegler, Matthias Brendel

**Affiliations:** 1 Department of Nuclear Medicine, University Hospital LMU Munich, Marchionini Strasse 15, 81377 Munich, Germany; 2 German Center for Neurodegenerative Diseases (DZNE), Feodor-Lynen-Strasse 17, 81377 Munich, Germany; 3 Munich Cluster for Systems Neurology (SyNergy), 81377 Munich, Germany

**Keywords:** refinement, enrichment environment, reduction, small animal positron emission tomography, longitudinal study, Alzheimer disease

## Abstract

**Simple Summary:**

Animal models continue to be necessary in many research fields, accompanied by ongoing ethical discussions regarding animal welfare. Therefore, we describe, in detail, our daily practice focused on the improvement of animal welfare (such as handling, enriched environment, study design, and experimental procedures), which results in a weight gain over time that has been shown to be an indicator of well-being. We also describe the reduction in the number of animals needed for our projects, thanks to the establishment of longitudinal studies.

**Abstract:**

Longitudinal studies on mouse models related to Alzheimer disease (AD) pathology play an important role in the investigation of therapeutic targets to help pharmaceutical research in the development of new drugs and in the attempt of an early diagnosis that can contribute to improving people’s quality of life. There are several advantages to enriching longitudinal studies in AD models with Positron Emission Tomography (PET); among these advantages, the possibility of following the principle of the 3Rs of animal welfare is fundamental. In this manuscript, good daily experimental practice focusing on animal welfare is described and commented upon, based on the experience attained from studies conducted in our Nuclear Medicine department.

## 1. Introduction

Alzheimer disease (AD) is a debilitating disease that causes progressive decline in cognitive and motor function, significantly reducing quality of life [1]. It is characterized by an early decrease in brain glucose metabolism [2], as well as the presence of amyloid plaques and neurofibrillary tangles. In addition, histological studies demonstrate that neuroinflammation is also a key feature of the AD brain [3]. Amyloid plaques are surrounded by activated astrocytes that produce reactive oxygen and nitrogen species, which may contribute to AD [4]. Animal models of AD are useful for studying these changes and the progression and development of the disease, with the goal of finding new diagnostic and treatment strategies. As the factors involved in the development and progression of the disease are different depending on the time during which the disease starts to manifest, different animal models for longitudinal studies are available.

Each model reflects biological features related to AD. Involvement and the effects of all these factors during the time of disease development are of high interest. Positron emission tomography (PET) is a molecular imaging method offering a large variety of radioactively labelled substances targeting different biological structures or processes. It offers non-invasive measurement of the radiotracer concentration in tissue and is clearly translational, since the exact same tracers and imaging technology can be applied in animal models, as well as in research and clinical use in humans. For example, PET remains one of the few methods to allow direct assessment of the human central nervous system (CNS) pharmacology, providing information on target engagement and supporting dose selection [5]. Thus, it has an increasing role in studying the biochemical and physiological dynamics of the CNS. The goal of the 3Rs Principle [6] is to avoid animal experiments (Replacement), to limit the number of animals (Reduction), and to limit their suffering in tests to an absolute minimum (Refinement). From the perspective of the design and development of a longitudinal study, the principle of the 3Rs plays a fundamental role in the research of all the strategies aimed at maintaining animal welfare for the entire time of the experiment. Due to their shorter lifespan, life-course results are obtained much more quickly in animal models, which is especially important when studying aging and transgenerational disease transmission [7]. Unfortunately, the complex interactions between organs and cells within their regular environment still necessitate the use of animal models in some cases. For example, in order to establish cause–effect relationships, connections between systems and the onset of age-related pathologies need to be studied. Since animal experiments cannot be completely replaced in our research on the development of AD, we implemented different strategies, following the principles of Reduction and Refinement. Improving the quality of life for experimental animals and reducing their stress and pain are among the goals that must be considered when it is not possible to replace animals, according to the principle of the 3Rs. EU Directive 2010/63 states that the animals should have “space of sufficient complexity to allow expression of a wide range of normal behavior” [8] (Annex III, Section A, paragraph 3.1.). For this reason, we aim for the enrichment environment we use to reflect the normal living conditions of the animals as much as possible. Currently, the term enrichment environment (EE) refers to various objects, or a combination of them, that can be added to the bedding and nest material (which are now considered basic components). These components play the roles of cognitive, sensory, social, and motor stimulators, which promote the interaction of animals with objects and with each other [9]. The scientific literature is full of examples of benefits due to EE. For example, it can prevent barbering [10], it reduces the likelihood of alopecia [11], and it decreases the expression of abnormal repetitive behaviors and anxiety [12], as well as the development of depressive-like phenotypes [13]. We can also assume that EE prevents or mitigates the onset of boredom-like symptoms in mice, since, in humans, this can be triggered by predictability, monotony, and confinement, and similar phenomena in rodents indicate that boredom in laboratory animals is real [14]. Considering the gender difference, groups of males in an environment with excessive EE demonstrated a higher occurrence of aggression [15]. On the other hand, without adequate EE, the conditions of their natural habitat would not be reproduced (spread out, keep away, escape, hide, and predict the occurrence of aggressive encounters). In order to prevent aggressive events in male mice, in our facility, they are always located in a cage with littermates with the same genotype, or with around one week of age difference with the same genotype, as it is known that this can prevent aggression and, consequentially, the necessity of single housing [16]. In this report, we summarize methods of ensuring animal welfare in longitudinal studies characterizing age-related processes in mouse models of AD. In addition to the non-invasiveness of PET imaging, a number of established techniques have been adapted, which contribute to the 3Rs in repeated measurements.

## 2. Materials and Methods

### 2.1. Animal Models

In our longitudinal studies over recent years, we used the following mouse models:

- APPSL70 mice, transgene of the Amyloid Precursor Protein (APP) in Swedish and London mutations [17] (Figures 5–8 Section 3.1, Section 3.2 and Section 3.3) (*n* = 92, half group was treated);
- APP-NL-GF mice knock-in model for the APP gene in Swedish, London, Arctic, and Iberian mutations [18] (Section 3.2) (*n* = 79);
- APPPS1 and PS2APP mice, mutated, respectively, for the APP gene and for the genes of presenilin 1 (PS1) and 2 (PS2) [19] (Section 3.2) (*n* = 46 and *n* = 36);
- APPPS1 X Trem 2 mice, transgene of APP, as well as of PS1 for triggering receptor expressed on myeloid cells type 2 (TREM2) [20] (Section 3.2) (*n* = 33);
- P301S mice, mutated for the microtubule-associated protein (TAU), which is abnormally aggregated in neuronal and glial cells in AD [21] (Section 3.2) (*n* = 46);
- C57BL/6J wild–type mice (WT) as control group (Section 3.2 and Section 3.3) (*n* = 46).

In the present study concerning the principle of the 3Rs applied to longitudinal studies in AD research, the APPSL70 is the mouse line used the most for examples.

### 2.2. Study Design

**Figure 1:**
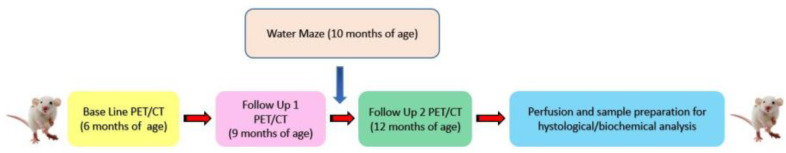
Study design of longitudinal AD studies.

We performed longitudinal studies in AD mouse models, in which each animal acted as its own control. The models consisted of PET/CT scans from 3 to 5 time points, with 2 to 4 different radiotracers characterizing changes in biological targets up to the age of 12 to 18 months. Furthermore, a Morris water maze (MWM) test was integrated before or after the last PET/CT time point in order to study spatial learning. At the end of each study, an intracardial perfusion with PBS was performed after intraperitoneal injection of a solution of 300–500 µL (weight-dependent) ketamine/xylazin (4 mL Ketamine 10%/1 mL Xylazine 20 mg/mL from Serumwerk Bernburg Germany, up to 24 mL with NaCl 0.9%), for the preparation of samples for histological and biochemical analyses (Figure 1).

### 2.3. Positron Emission Tomography

In each animal, we used 2 to 4 of the following ^18^F labelled tracers: [^18^F] D2-Deprenyl for reactive astrocytes which surround the Beta Amyloid plaques [3]; [18F] Florbetaben for beta amyloid accumulation; [^18^F] Ge-180 for the 18-kDa translocator protein (TSPO), as its local upregulation is a sensitive marker for the microglial activation in AD brains [22]; and [^18^F] UCB-H for synaptic density (synaptic loss or synaptic sprouting) [23]. PET imaging was performed under constant anesthesia with isoflurane (1.5% at 1.5L oxygen flow per minute) with a Nanoscan PET/CT (Mediso Ltd., Budapest, Hungary). For anatomical information, the system was equipped with an X-ray Computed Tomography System (CT) in line with the PET scanner. After induction of anesthesia with isoflurane, the eyes were protected from drying out by topical application of an eye ointment (Bepanthen, Bayer AG, Leverkusen, Germany). In order to judge the depth of anesthesia before the radiotracer´s injection, surgical tweezers were used to check whether the inter-toe reflex could still be triggered. If it could no longer be triggered, a micro-catheter was inserted into the lateral tail vein (30 G needle, 7 cm plastic tube, 30 G attachment, flushing with 0.9% isotonic saline solution); the correct position of the indwelling venous cannula for the application of the radiotracer was checked by administering a small amount of isotonic saline solution (20–30 µL) into the catheter. The injected radioligand solution consisted of a total of 150 µL, and contained approximately 20 MBq of radioactive tracer. PET/CT measurement was carried out up to 60 min post injection: for [^18^F] Ge-180 and [^18^F] Florbetaben, the measurement was carried out for 30 min., 60 and 30 min. after radiotracer uptake, respectively (static scan); those for [^18^F] D2-Deprenyl and [^18^F] UCB-H were carried out immediately post injection, for 60 min (dynamic scan). Up to four animals were measured in parallel, and image data were generated for each animal from head to tail. After the experiment, the mice were placed in a fresh temporary cage with food and water, warmed by heating mats. The animals were returned to their home cages only when they were fully awake.

The radioactive waste coming from the animals (feces, urine) and all the equipment used on the workspace (e.g., syringes, paper tissue, paper mats, and gloves) were collected in black boxes, upon which were written the isotope used and the date and time of the experiment. These boxes were then collected by the radiation safety personnel of the department.

### 2.4. Water Maze

The Morris Water Maze (MWM) is useful to test hippocampal-dependent learning, including acquisition of short- and long-term spatial memory [24]. Typically, it consists of a six-day trial, and it has to be conducted by the same operator in the same room in order to reduce odor trail interferences [25]. In brief, the first test day served for acclimatization to the visible platform (5 min per mouse). Thereafter, the mice underwent five training days, during which each mouse had to perform four trials per day, with the platform visible on the first training day, and the platform hidden under water for all other training days. After the trial, the mice were placed in a heated box to dry. The test day entailed a single trial with complete removal of the platform. The trial length on all training and test days was set to a maximum of 70 s. The video tracking software EthoVision® XT (Noldus) was used for analyses of escape latency, platform frequency, and attendance on the platform quadrant during the trial. In our longitudinal studies, the test was conducted in a room adjacent to both the cage facility and the room where the preparation and PET scans took place, and the mice resided in a cabinet in the same behavioral room for the duration of the trial. This minimized the stress caused by this additional procedure and by moving animals to other, more distant, locations. The personnel who carried out the MWM test received appropriate training from a veterinarian before the experiment and was also part of the team that regularly deals with animal welfare, as well as with the preparation and execution of PET scans.

### 2.5. Enrichment Environment

**Figure 2:**
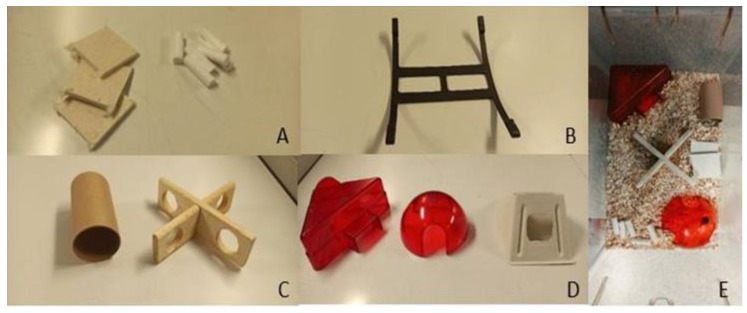
Basic nest (**A**), double mouse swing (**B**), paper or plastic tunnel and wooden cross with holes (**C**), and mouse angle house, igloo, and paper angle (**D**). Example of cage with EE used in our studies (**E**).

In our facility, the male mice are housed (in groups of 3–4 siblings, with one week age difference and same genotype) with the same EE as the females. They are more intensively monitored (for too much grooming, self-isolation attributable to the attempt to escape from the attacks of the other mice, and the presence of wounds) so as to prevent the onset of high levels of aggression, but still keeping them in a condition to be able to maintain a good level of play and sociopositive behavior. The female mice are housed in groups of 3 to 5. All of the mice are housed in large IVC-Techniplast cages (425 mm × 266 mm × 185 mm), with a 12:12 h light: dark cycle, humidity of 45–65%, and temperature of 23–26 °C. All toys are changed once a week. The food (standard diet from Sniff Spezialdiäten GmbH) is placed ad libitum, as is water. Mice were allowed to acclimatize to their environment for 7 days before any experimental procedure. The EE can be divided into categories (Figure 2) [26]. In our studies, we used the basic nest (2A), structural EE (2B), foraging EE (2C) and housing EE (2D):

### 2.6. Handling

**Figure 3:**
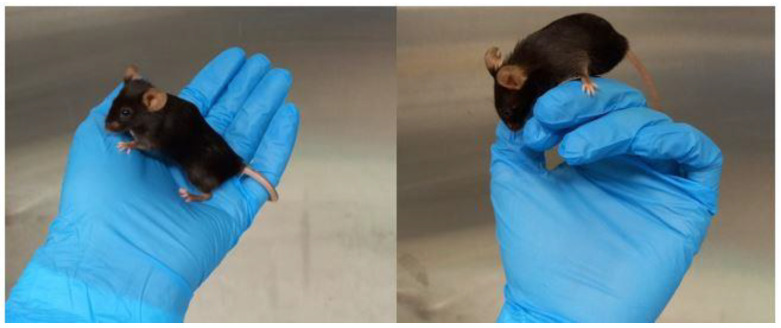
Gentle handling applied for all experimental procedures. As can be seen, the mouse shows normal exploration behavior.

We use a handling method that reduces stress, and it can be used both during experiments and in daily practices (such as weighing, animal checks, and changing cages) and does not need any additional material (for example, tunnels). Once the animal is picked up by the tail from the cage, with a quick but gentle twist of the hand, it is immediately placed in the cupped hand (Figure 3).

### 2.7. Monitoring Signs of Stress

**Figure 4:**
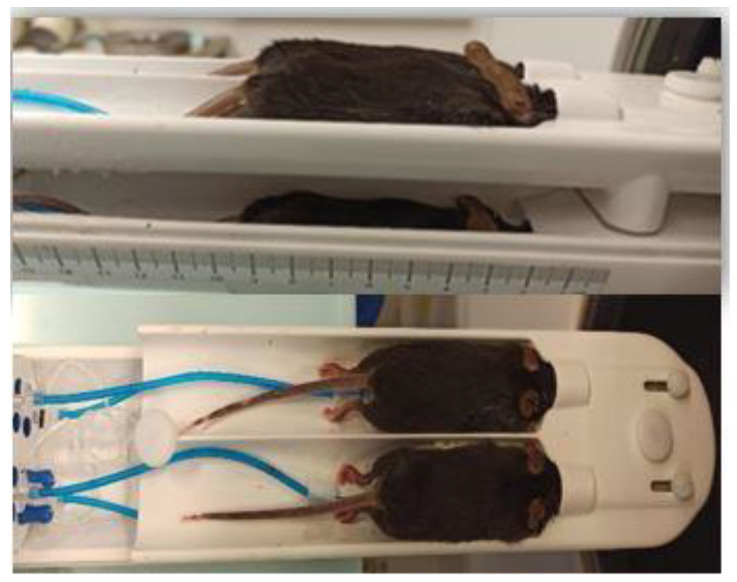
Four-mouse Hotel Bed (Mediso, Budapest, Hungary), in which two mice are positioned on the lower part and two on the upper part. The parts are connected, and the isoflurane and oxygen can easily and continuously be administered to the mice for anesthesia. Underneath the mice, there are small pillows (at the end of the blue tubes, under the mice’s chests) that monitor the respiratory rate.

Animals are monitored for any sign of stress using the Mouse Grimace Scale. It consists of a standardized behavioral coding system with high accuracy and reliability in which no-pain pictures and descriptions are compared with pictures of moderate and severe distress via mouse facial expression (e.g., orbital tightening, nose bulge, cheek bulge, ear position, and whisker change) [27]. In our longitudinal studies, the mice are scanned two or, maximum, three times a week for each time point, and they are always observed in the postanesthesia phase, until they have completely recovered. They are also checked on the day after the procedures according to the Mice Grimace Scale, in order to identify the onset of visible postprocedural stress symptoms. It is known that this scoring system allows for the identification of the degree of pain in mice, but also shows that they manifest their pain using facial expressions [26]. Furthermore, the MGS measures not only pain, but also distress, fear, and discomfort, which can be detected by the ear and eye score [28]. Mice hide signs of pain and suffering to avoid becoming prey, and, generally, they show only subtle signs of suffering and pain, such as weight loss [29]. In addition to the parameters shown in Figure 4, the weight of each mouse is monitored up to once per week during all the experimental phases, as it has been demonstrated that mice gain weight when the stress level is lower, and this can be achieved with appropriate EE and enough ventilation (in IVC cages) [30].

### 2.8. Anesthesia

In contrast to human studies, the imaging of small animals generally requires anesthesia [31]. In our longitudinal studies, we use isoflurane because it is well suited for animal PET scans for up to 6h of measurement time [32]. Repeated isoflurane anesthesia causes only mild short-term distress and impairment of well-being, mainly in the immediate postanesthetic period [33]. This can be kept under control by monitoring the respiratory rate and heart rate, and keeping the body temperature of the animals constant throughout the process in order to prevent the onset of hypothermia. Maintaining the temperature and anesthesia at a constant level during the experiment minimizes any potential pain, suffering, distress, or lasting harm to the animal [34]. In a study conducted by Baier J et al. in 2020 [35] on repeated MRI scans, using isoflurane for general anesthesia and its maintenance three times a week for four weeks, the mice showed no alterations in animal welfare due to the repeated procedures. Another study [36] demonstrated that nest-building activity is not altered by a second exposure to isoflurane.

### 2.9. Mouse Hotel Bed

Using a four-mouse bed (Mediso Ltd., Budapest, Hungary) it was possible to scan multiple mice (up to four) simultaneously (Figure 4). The use of this chamber is advantageous in many aspects:

1. The chance to perform more scans in the same day using radioactive isotopes with a short half-life (^18^F: 109, 7 min). This is especially important in longitudinal studies with a large number of animals because all of the scans have to be performed respective of the age-related time point.
2. There is a lower injected volume. This is important, as the ^18^F-based tracers decay quickly. Since the activity has to be the same for each animal, the volume of the injection needs to be increased according to the radioactive decay in sequential scans. Thus, the volume to be injected would be above the maximum limit allowed for these animal experiments. Using the 4-mouse bed, this problem is circumvented, since four mice are injected and scanned at the same time (instead of four individual scans one after the other).
3. This particular bed for four animals is equipped with a heating system that keeps the temperature of the bed constant at about 37 °C, which is very important during anesthesia, as hypothermia can arise, and also because the temperature can have an effect on the biodistribution of the injected radiotracer. In addition, there is a monitoring system for cardiac and respiratory function that allows continuous monitoring in each animal.

## 3. Results

### 3.1. Longitudinal Studies Enable the Analysis of the Same Animal with Different Radioligands during Progressive Aging

**Figure 5:**
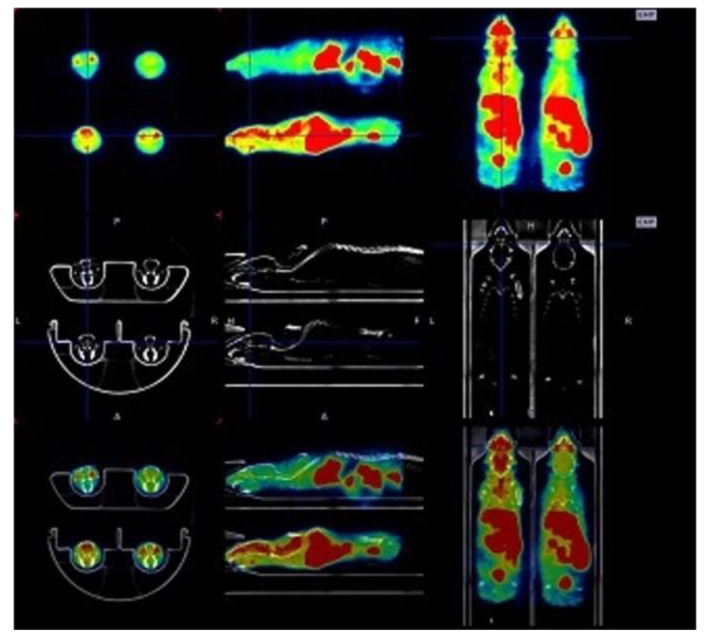
From top: example of PET, CT, and FUSION PET/CT of four mice imaged simultaneously using the four-mouse bed. The four APPSL70 mice were scanned for 30 min after 30 min uptake with 20 MBq [^18^F] Florbetaben tracer for beta amyloid accumulation. Axial, sagittal, and coronal slices.

Figure 5 shows an example of PET, CT, and Fusion PET/CT of four mice imaged simultaneously.

**Figure 6:**
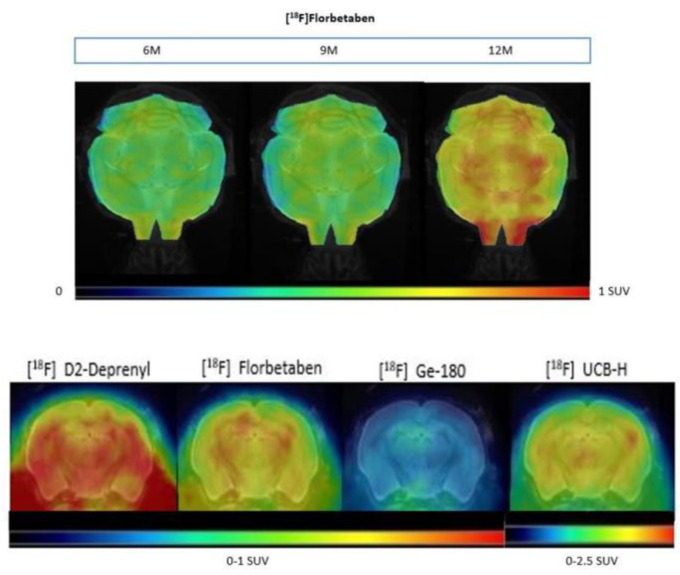
**Top panel:** Example of an APPSL70 mouse brain in standard uptake value (SUV) scale. PET projection view and MRI reference with the same ^18^F radiotracer ([^18^F] Florbetaben) at 6, 9, and 12 months of age (left to right). **Lower panel:** Coronal slices of PET scans of a transgenic AD-related mouse brain at 12 months of age after injection of four different radioligands.

Figure 6 shows an example of beta amyloid in a transgenic mouse brain over time with the same radiotracer ([^18^F] Florbetaben): the same mouse was scanned at 6, 9, and 12 months of age. Coronal slices of the brain from the same animal after the injection of four different radioligands at 12 months of age with a reference MRI (Magnetic Resonance Imaging) used as anatomical reference are also included.

### 3.2. The Longitudinal PET Studies follow the Principle of Reduction by Russell and Burch

**Figure 7:**
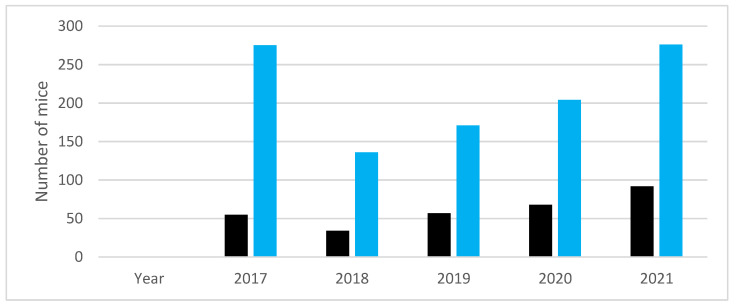
Difference between number of mice used (in black) and the number that would have been necessary, in theory, if separate groups had been used in longitudinal studies (in blue). The data reported include the AD models APPPS1, APPPSL70, P301S, APPPS1XTrem2, APP-NL-GF, PS2APP, and C57BL/6 control mice.

The graph in Figure 7 shows the number of mice used by our research group in longitudinal studies in recent years, and the number of mice that would have been necessary, in theory, for the same projects using separate groups for each time point.

### 3.3. Gentle Handling and EE Reduce Stress in Longitudinal Studies

For all the experiments, the MGS resulted in a score of zero.

**Figure 8:**
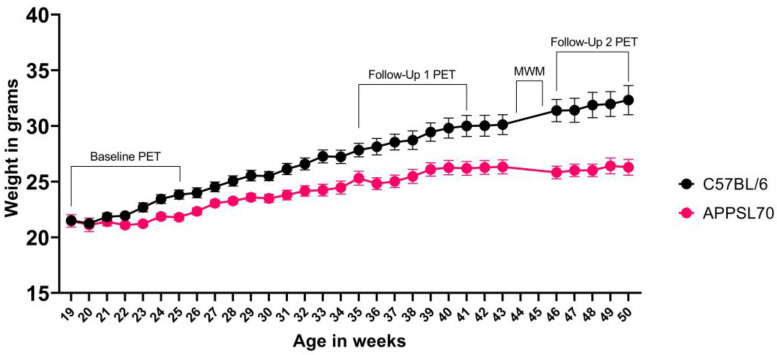
Weight/time graph during the longitudinal study with mice up to one year of age. Shown is the average weight (±STD) of C57BL/6J wild-type and APPSL70 AD mice. The time points within which the PET scans and MWM were performed are marked (APPSL70 *n* = 28, WT *n* = 17). These animals did not receive any pharmacological treatment.

We observed that, during the entire stay of the animals in the facility (9 months or more), the nest material was regularly used, as were the other components of the EE. The combination of gentle handling and EE makes the animals more docile and less stressed, even during the time before/during/after the experimental procedures. The graph in Figure 8 shows the development of the weight in a female AD mouse model (APPSL70, *n* = 28) and in wild-type controls (*n* = 17), each receiving PET scans with four different radiotracers at three different time points, with a MWM test before the last time point. We observed an average of 13% mortality during this experiment of up to six months, independent of the mouse line.

While the body weight of wild-type mice increased continuously, the body weight in the APPSL70 AD model did not increase further after ~10 months of age. A very similar weight gain (9.6% mean percentage of gain weight from 3 to 6 months of age and 11.8% from 6 to 9 months of age) was observed in the APPPS1 group, which consisted of 11 animals. No mouse, either wild-type or transgenic, had to be excluded from the trials due to stress/fear factors, and no animal developed signs of anxiety/stress over six months of study (up to one year of age for mice).

## 4. Discussion

We report on aspects of implementation of the 3Rs Principle in longitudinal mouse studies for AD research. Of note, the reported methods and findings concerning handling and housing are not restricted to AD research. Repeated PET/CT measurements, combined with a behavioral test, proved feasible in AD mouse models and wild-type mice without losing animals due to increased stress, up to 12–18 months of age. Reduction in the number of animals used for experimentation is achieved by longitudinal, multi-tracer PET scans in the same animal; thus, each mouse is its own control, and the results of behavior tests can also be correlated for each individual animal. No episodes of severe weight loss were detected during the study (according to our approved scoring system). The weight loss observed in the APPSL70 AD model is compatible with the findings in previous studies on different AD mouse models, showing that weight loss is connected to the reduction in body adiposity, an increase in energy expenditure, and a decrease in food efficiency connected to the progression of AD [37,38]. It is known that stress has a negative effect on the results of the MWM [39,40], and in some studies, it has been hypothesized that the stress from less frequent handling before the test can be responsible for the failure of some mice in finding the platform [41]. It is also known that the induction of stress produces learning deficits in the MWM [42,43]. In this context, considering that routine handling influences animal welfare and the relationship that is established between the handler and the animal, we observed that the non-stressful handling method assures low variability in the results obtained from the test. In all procedures, we adhered to the same animal handling procedure. The standard method, picking up the mice by the tail, induces more anxiety and stress compared to more gentle methods, such as cupping and tunnel handling [44,45]; it was also previously demonstrated that gentle handling can foster a better relationship between the handlers and the rodents, and, if implemented as the standard of care, handling can reduce depressive symptoms in mice, producing data that are more reliable and, in general, improve the animals’ well-being [46]. Ueno et al. [47], in 2020, demonstrated that repeated exposure of the mice to the experimenter´s hand before conducting behavioral tests allows them to become accustomed to it and to reduce anxiety about high altitudes. In addition, it has been previously shown that reducing mouse anxiety due to handling contributes to a reduction in the number of animals required for experiments [47]. The same handling method (described in Methods 2.6) is used to put the mice under general anesthesia before the radiotracer injection. Thus, no immobilization method is necessary. How much our long-term gentle handling (up to six months stay in the facility) effectively reduced stress in both male and female mice will need to be shown in a further study including non-gentle handling control mice. While there are several advantages to using the four-mouse hotel chamber for PET imaging, it has the limitation of keeping one single flow rate for all animals. Thus, individual adaption is not possible, but could be improved in a new design. Providing enriched environments in sufficiently large cages also contributes to decreasing mortality, as has been demonstrated by comparison to conventional housing [48]. We used established, qualitative measures of stress, as well as animal weight, as a surrogate of well-being. Quantitative measures, such as corticosterone level, could not be integrated into the study protocol, since the mice are sacrificed only at the end of the project, in order to follow the Reduction Principle.

## 5. Conclusions

In the context of longitudinal studies, animal welfare assumes particular relevance, since the animals reside for long periods in the facility during their progressive aging and, at the same time, are involved in all of the experimental phases. Non-invasive imaging using PET allows repeated measurements of multiple biologically relevant tracer distributions over time in the same animal, thus significantly reducing the number of animals required. Reduction is achieved with the establishment of longitudinal studies that use radioactively labelled molecules in very small-volume solutions without pharmacological effect. Refinement is ensured by the possibility of conducting PET/CT scans with four animals at a time, a varied and stimulating enriched environment, and a standardized handling method during all phases of the experiment with a minimal number of experimenters.

## Data Availability

Data presented here will be provided by the authors upon request.
